# Population status and genetic assessment of mugger (*Crocodylus palustris*) in a tropical regulated river system in North India

**DOI:** 10.1038/s41598-024-57983-2

**Published:** 2024-03-28

**Authors:** Surya Prasad Sharma, Mirza Ghazanfarullah Ghazi, Suyash Katdare, Ruchi Badola, Syed Ainul Hussain

**Affiliations:** Wildlife Institute of India, Chandrabani, P.O. Box # 18, Dehra Dun, 248002 Uttarakhand India

**Keywords:** Adaptive management, Ecological generalist, Population trend, Occupancy, Species recovery, Conservation translocation, Freshwater ecology, Restoration ecology, Ecology, Genetics

## Abstract

For rewilding the depleted crocodylian populations in India, a targeted ‘one-species one area’ based conservation approach was adopted in the early-1970s. Suitable habitats were identified and designated as protected areas, specifically targeted to recover a particular crocodylian species. A ~ 610 km stretch of Chambal River in the Ganga River Basin was declared as National Chambal Sanctuary to restore the ‘Critically Endangered’ gharial (*Gavialis gangeticus*), where active management of mugger (*Crocodylus palustris*) was discouraged. In the present study, we examined the population trends, occupancy, and genetic status of mugger by conducting population monitoring and genetic assessment to understand the status of potentially competitive mugger in the Sanctuary. Our finding suggests that the mugger population has notably increased and colonised the Sanctuary. We observed a moderate level of genetic diversity in the mugger, which was relatively higher compared to the gharial in the Sanctuary. The rapid colonization of ecological generalist mugger raises concerns about potential competition with ecological specialist gharial threatening its long-term sustainability. Considering the coexistence dynamics between the species, it is essential to extend adaptive management strategies for mugger to ensure successful recovery of gharial population in the Sanctuary.

## Introduction

Over the last few centuries, ecosystem degradation led by anthropogenic activities has triggered massive biodiversity loss resulting in demographic declines, range contractions, population isolations, and extinction of numerous species^1–3^. In response to this declining trend, micro-scale conservation measures such as species translocation have become imperative and integral for species restoration and rewilding to buffer the impact of human-driven biodiversity loss^4–6^. In the past, translocation efforts were primarily motivated by the desire to establish or enhance populations of target species for various purposes like food resources, pest control and trophy hunting^7–9^. Improved scientific understanding led to recognition of ecological implications of species translocations, promoting its global adoption as a conservation strategy and substantial increase in species translocation programs globally^10,11^. Species translocation has successfully prevented extinction of several wildlife species, such as the Arabian oryx (*Oryx leucoryx*)^12^, Californian condor (*Gymnogyps californianus*)^13^, and Lord Howe Island woodhen (*Hypotaenidia sylvestris*)^14^.

By the late-1960s, the crocodylian populations in India had also witnessed a significant population and range decline primarily attributed to habitat loss caused by sand mining and agriculture, poaching, and mortality in passive and detrimental fishing^15,16^. To address this situation, the government of India listed all three crocodylians i.e. gharial (*Gavialis gangeticus* Gmelin, 1789), mugger (*Crocodylus palustris *Lesson, 1831), and saltwater crocodile (*Crocodylus porosus *Schneider, 1801), in the Schedule I of the then enacted Wild Life (Protection) Act, 1972, prohibiting their hunting, and trade in any form^17^. Subsequently, a dedicated crocodile conservation and management program, commonly termed ‘Project Crocodile,’ was launched in 1975 and aimed at protecting the remaining natural populations of crocodylians by creating new protected areas, enhancing population recruitment and recovery through a 'head-start’ assisted conservation translocation, and establishing captive-rearing and breeding facilities within species range^18^. In a decade of its inception, more than 12 Wildlife Sanctuaries were created under the Wild Life (Protection) Act, 1972^18,19^. Simultaneously, rearing and captive breeding facilities were established, and crocodylians of wild origins were captively reared in these facilities and later released into the protected areas^16,20,21^.

The mugger is widespread in India and occurs sympatrically with gharial especially in the Northern and Eastern Indian rivers^22^. Due to the sympatric occurrence and likelihood of interspecific competition, a targeted 'one-species one area’ based conservation approach was suggested^19,20^. Following which, there has been a scientific consensus to restrict the release of mugger in the riverine sanctuaries dedicated for gharial conservation^17,20^. Consequently, gharial specific conservation management interventions were implemented in the riverine protected areas viz*.* National Chambal Sanctuary (NCS) in the Chambal River, Katerniaghat Wildlife Sanctuary in the Girwa River, Son Gharial Sanctuary in the Son River, and Ken Gharial Sanctuary in the Ken River of the Northern region and Satkosia Gorge Wildlife Sanctuary (SWGS) in the Mahanadi River of the Eastern region^18,23^. Whereas, conservation and management efforts for the mugger were primarily concentrated in selected protected areas viz*.* Krishnagiri Wildlife Sanctuary*,* Coringa Wildlife Sanctuary, Papikonda Wildlife Sanctuary and Manjira Wildife Sanctuary in the southern region of India^19,22^.

Conservation management has facilitated the rapid recovery of the severely depleted gharial populations in the riverine protected area^16^. The gharial population in the Chambal River has increased from 107 individuals in 1979 to approximately 1675 individuals in 2018^15,24^. In subsequent years, despite the initial precautionary measures taken, the lack of effective communication and coordination has resulted in inadvertent release of mugger individuals into the NCS and SGWS^21,23^. The increase in mugger population in the SGWS has resulted in interspecific aggression and conspicuous dominance of mugger^25^. The dominance of mugger in the SGWS also been attributed to the non-survival of the gharials in the past^23^. The increase in mugger population in prime gharial habitat, combined with depleting resources may intensify the competition and disrupt the coexistence dynamics^26,27^, undermining decades long gharial conservation efforts. Hence, monitoring of sympatrically occurring mugger is essential for guiding the gharial recovery program and develop a comprehensive framework for maintaining the coexistence between theses sympatric species in a single riverscape^28^.

In the present study, we intend to address the following research questions to understand the status of mugger population in the National Chambal Sanctuary in the Chambal River, (a) What is the present population trend of the mugger? (b) What is the extent of occupancy of the mugger, and what factors (habitat, ecological and anthropogenic) influence its occupancy? (c) What is the genetic status of the mugger population in the Chambal River?

## Results

### Population trends

We observed a consistent increase in the numbers of mugger crocodiles over time, as indicated by the relative density index (RDI) (Supplementary Fig. 1). The adult and sub-adult size classes constituted a significant portion, ranging between 81 and 84% of the population. Conversely, the percentage composition of the juvenile and below-size classes varied between 0 and 8% (Supplementary Table 1). This observation highlights the presence of different age cohorts within the mugger population. The exponential rate of increase (r) during the period was 0.101 (F—901.07; P < 0.001; R^2^—0.98) (Supplementary Fig. 2). The annual finite population growth rate (λ) was 1.106 (95% CI = 1.099–1.113). The percentage change estimated from λ was 10.6% (95% CI = 9.9–11.3%) per year (Table 1).

**Table 1:** The population growth trend of the mugger population between 2003 and 2019 in the National Chambal Sanctuary, India.

Methods	2003–2019
Exponential rate of increase (r)	0.101(0.094–0.107)
Finite population growth rate (λ)	1.106 (1.099–1.113)
Percentage change in population	10.6% (9.9–11.3%)

The confidence intervals (95% CI) are shown in parentheses.

### Single-season site occupancy

The estimated naïve occupancy for the mugger in the 134 composite blocks was 0.88 (± 0.04) (95% CI = 0.80–0.96). The univariate model containing channel width yields the lowest Akaike Information Criterion (AIC) for the probability of detection. Further, the additive models predicted five best models under ΔAIC ≤ 2, and the AICwt ranged between 0.12 to 0.28 (Table 2). The covariates associated with the best-predicted model (ΔAIC = 0) were river width for detection (p), and channel depth and sand for occupancy (Ψ). The goodness of fit test of the most parameterized multivariate occupancy model showed no evidence of overdispersion or underdispersion. The best model (ΔAIC) contained three covariates: river width, depth, and sand. The covariate river width has a negative influence on detection (p), and both site-covariates, viz*.*, river depth and sand, positively impact occupancy (Ψ) (Table 2 and Supplementary Table 2). The estimated 95% confidence interval of the beta coefficient [β ± 1.96 × SE (Standard Error)] was used to determine the impact of covariates on detection and occupancy. Two covariates, river width for detection and river depth for occupancy, yielded strong support with non-zero overlapping. The river width had non-zero overlapping estimates in all candidate models (ΔAIC ≤ 2). In contrast, river depth had non-zero overlap in four out of five candidate models for influence on occupancy (Supplementary Table 2). In contrast, other covariates did not obtain much support for impact on mugger detection and occupancy.

**Table 2:** The results of model selection for determining mugger detection(p) and occupancy (Ψ) probabilities in the National Chambal Sanctuary, India.

Model	AIC	ΔAIC	AICwt	Chi	c-hat	P-value
p (width), Ψ (depth + sand)	532.3	0	0.28	4.6	0.84	0.54
p (width), Ψ (depth + mining)	532.8	0.47	0.22	4.3	0.84	0.54
p (width), Ψ(depth)	533.0	0.67	0.2	4.3	0.83	0.55
p (width), Ψ (depth + width)	534.1	1.76	0.12	4.2	0.78	0.59
p (temp + width), Ψ(depth)	534.1	1.78	0.12	4.4	0.82	0.57

The best variables for mugger detection and occupancy predictors were estimated using summed Akaike weights (ΣAICwt). The ΣAICwt for detection (p) was highest for river width (ΣAICwt—0.99) followed by air temperature (ΣAICwt—0.12), and for occupancy (Ψ), it was highest for river depth (ΣAICwt—0.99) followed by sand substrate type (ΣAICwt—0.28) and frequency of mining (ΣAICwt—0.22). The estimated mean model-averaged prediction for best candidate models was 0.57 ± 0.005 (mean ± SE) for detection and 0.88 ± 0.01 for occupancy (Supplementary Fig. 3).

### Genetic assessment

We successfully extracted DNA from 106 of the 122 samples (86%) and genotyped 81 of the 106 samples (76%) (Supplementary Table 2). The average amplification success across 10 polymorphic loci rates was 88.61 ± 1.44. The quality index was 0.93 ± 0.008 across the 10 polymorphic loci, and the average error rate per locus was 0.07 ± 0.008 (Supplementary Table 4). We found no evidence of a large allele dropout in our data, and the null allele frequency for each locus was below 5%. No single locus showed significant deviation from Hardy–Weinberg Equilibrium across all nesting sites. Therefore, all loci genotyped were used for further analyses.

**Figure 1:**
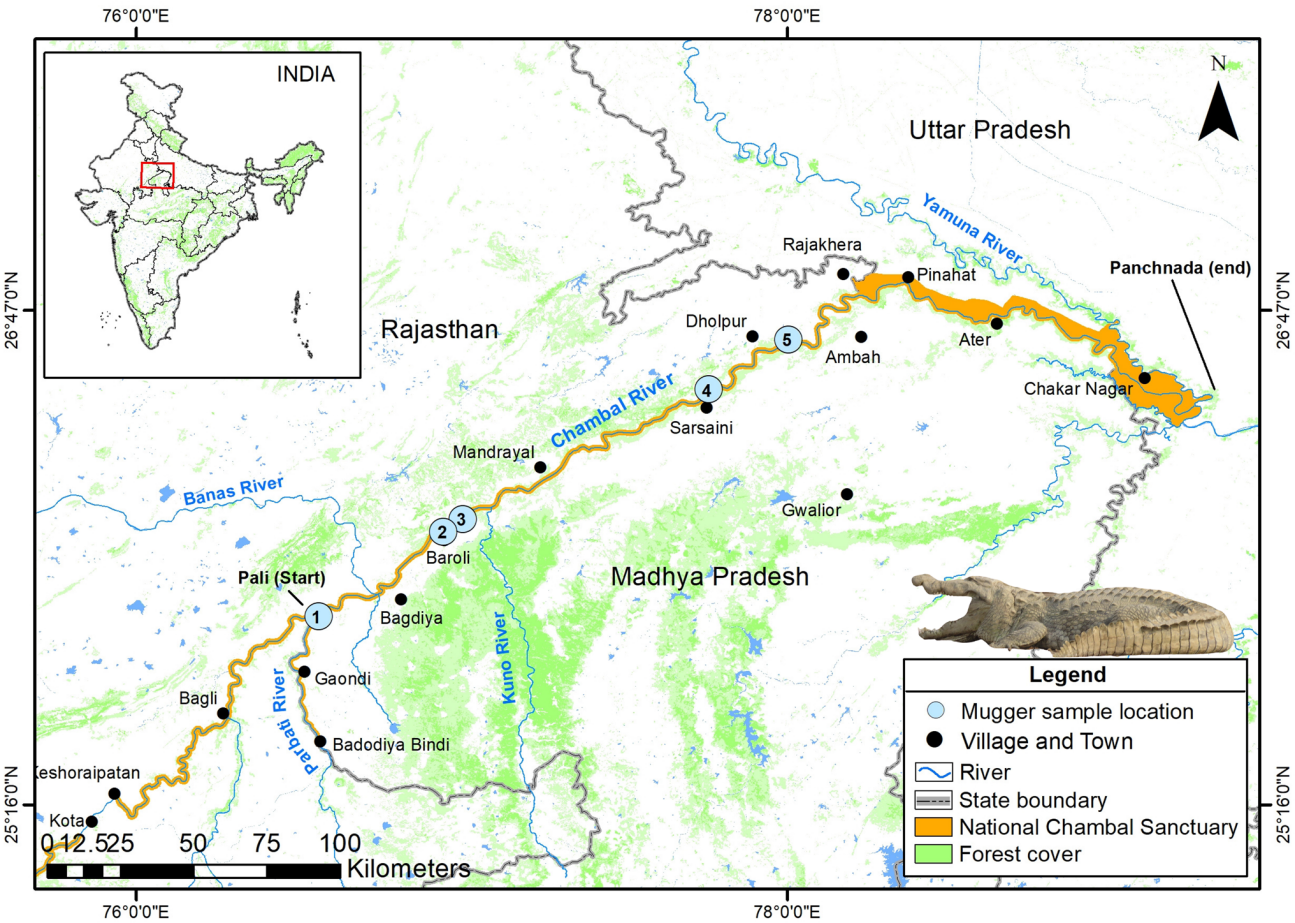
Map showing study area, survey stretches, and sampling locations for genetic analysis in the Nation Chambal Sanctuary along the Chambal River, India. The survey was conducted between Pali (start) and Panchnada (end) ~ 400 km stretch. The labeled sodalite blue represents sampling locations (1 = Pali, 2 = Baroli, 3 = Nadigaon, 4 = Dangbasai, and 5 = Tigri). The map was prepared using ArcGIS v.10.3.1 software developed by ESRI (https://www.esri.com).

The cumulative probability of identity PID or probability of identity, PID-unbiased was 9.93 × 10^–8^, and PID-sibs was 1.17 × 10^–3^ for a panel of 10 polymorphic loci. We identified 60 unique individuals from 122 biological samples collected across five mugger nesting sites using multilocus genotype data (Fig. 1). The Dangbasai (DG) nesting site had only one individual genotype; therefore, we excluded it from further analysis. The number of polymorphic loci varied across the nesting sites, with the Baroli (BR) nesting site showing the most (n = 10), followed by the Nadigaon (NG) (n = 9), Pali (PA) (n = 8), and Tigri (TG) (n = 8). The number of alleles observed at each locus ranged from 2 to 14. The overall mean number of alleles per locus was 5.2 ± 1.4 (Table 3). The BR showed the highest number of alleles per locus BR (3.9 ± 0.7), followed by NG (3.8 ± 0.8), TG (2.0 ± 0.6), and PA (2.1 ± 0.3). The allelic richness based on a minimum sample size of six individuals was 2.7 ± 0.7 (Supplementary Table 5). The overall observed (Ho) and expected (He) heterozygosities were 0.57 ± 0.08 and 0.55 ± 0.08, respectively.

**Table 3:** The genetic diversity estimates of 59 individuals of muggers in the National Chambal Sanctuary, India.

Locus	N	Na	Ne	Ho	He	F
CpPSSR14	58	3.000	1.783	0.397	0.439	0.097
CpPSSR12	59	2.000	1.052	0.051	0.050	− 0.026
CUD68	58	5.000	3.393	0.724	0.705	− 0.027
4HDZ27	59	3.000	2.635	0.780	0.621	− 0.256
4HDZ391	59	14.000	7.470	0.898	0.866	− 0.037
G13_2	59	4.000	2.564	0.780	0.610	− 0.278
G13_14	59	4.000	1.584	0.373	0.369	− 0.011
G13_18	59	2.000	1.955	0.475	0.488	0.028
CpP203	59	13.000	5.966	0.881	0.832	− 0.059
CpP208	59	2.000	1.972	0.407	0.493	0.175
Mean	58.800	5.200	3.037	0.576	0.547	− 0.039
SE	0.133	1.420	0.655	0.088	0.076	0.044

Number of samples (N), number of alleles per locus (Na), observed heterozygosity (Ho) and expected heterozygosity (He), and Inbreeding coefficient (F).

**Figure 2:**
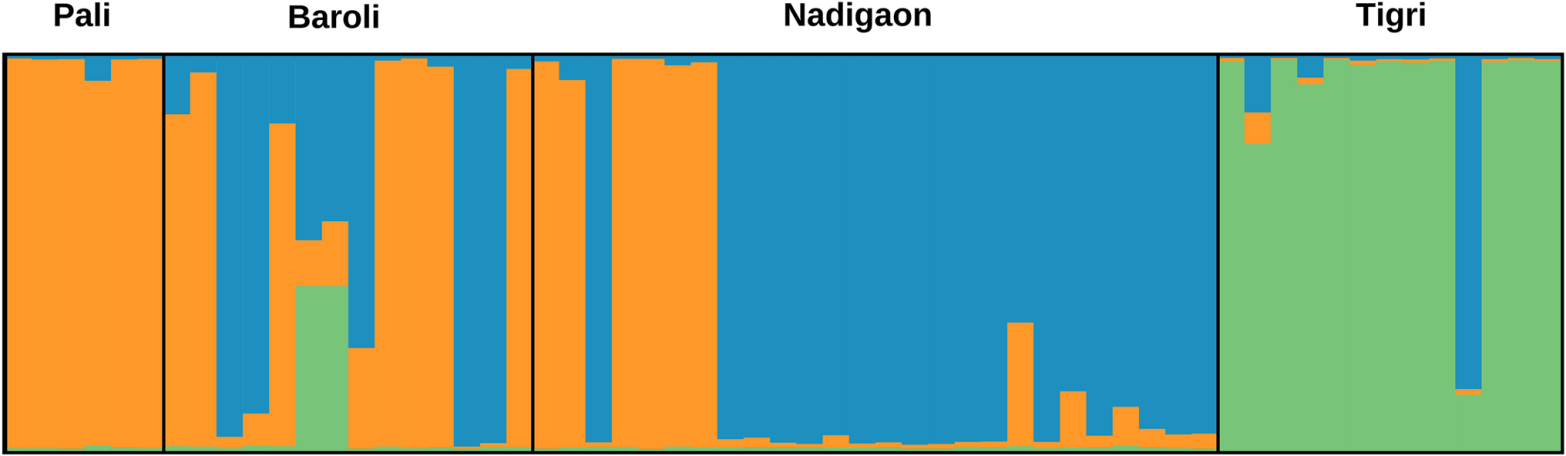
The Structure barplot of 59 mugger individuals from four nesting sites (Pali, Baroli, Nadigaon, and Tigri) in the National Chambal Sanctuary, India. Each bar indicates an individual, and the extent of color in the bar indicates the probability of assigning the individual to a particular cluster. The plot was prepared using Distruct v1.1 (https://rosenberglab.stanford.edu/distruct.html).

**Figure 3:**
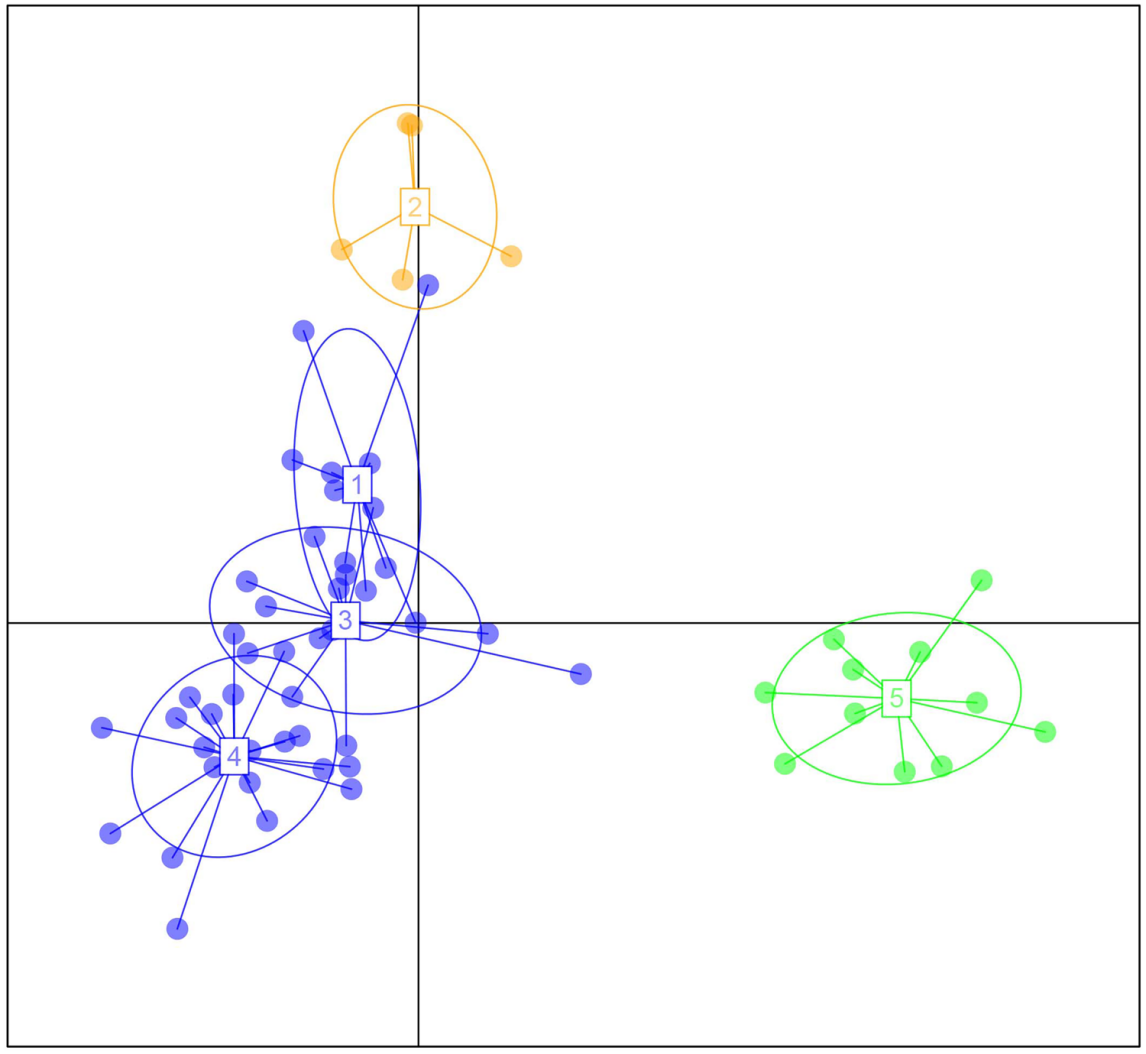
Discriminant Analysis of Principal Component (DAPC) scatterplot of 59 mugger individuals in the National Chambal Sanctuary, India. The scatter points represent individual observations, and each color represents a genetic cluster. The plot was prepared in RStudio 2023.03.1 (https://posit.co/download/rstudio-desktop/) using package *adgenet* (https://adegenet.r-forge.r-project.org/).

The Bayesian clustering analysis implemented in Structure v2.3.4 suggests the presence of three (K = 3) clusters inferred by the delta K estimates (Supplementary Fig. 4). All individuals sampled from PA were assigned to Cluster-I with an overall proportion of membership (q) equal to 0.975, and 92% individuals sampled from TG were assigned to cluster-III with q equal to 0.901 and 8% to Cluster-I. The individuals sampled from BR and NG were assigned to multiple clusters. (Fig. 2). The Discriminant Analysis of Principal Components (DAPC) identified five clusters (K = 5) using the Bayesian Information Criterion (BIC) (Supplementary Fig. 5), segregating them into three units (Fig. 3). We observed significant (P < 0.05) genetic differentiation measures between all sites except BR and NG. The estimated *F*_ST_ value ranged between 0.087 (PA-BR) and 0.286 (PA-TG), and Jost’s *D* value between 0.039 (PA-BR) and 0.188 (PA-TG) (Table 4). Both analyses showed presence of three genetic clusters within the sampled stretch (I-PA, II-BR/NG, and III-TG).

**Table 4:** The results of genetic differentiation parameters in the National Chambal Sanctuary, India.

Nesting sites	PA	BR	NG	TG
PA	–	0.039*	0.089*	0.188*
BR	0.087*	–	0.004	0.108*
NG	0.136*	0.012	–	0.147*
TG	0.286*	0.158*	0.172*	–

The above diagonal represents Jost’s *D* values, and the below diagonal represents *F*_ST_ values.

*Significant values P < 0.001.

The estimated *M* ratio across 10 polymorphic loci analysed was 0.33 ± 0.08, and the Heterozygosity Excess Test (HET) performed under both mutational models yielded significant (P < 0.05) values for heterozygosity excess (Stepwise Mutation Model P = 0.04; and Two-Phase Mutation Model P = 0.04). Both the approaches i. e., *M* ratio and HET, detected a signature of genetic bottleneck. We also observed a shift in allele frequency distribution mode. However, the pooling of samples from different clusters may have affected the result in performing bottleneck detection.

## Discussion

Conservation translocations have significantly averted the risk of pushing several threatened species to the brink of extinction^5,29^. Despite widespread acceptance and common use in achieving targeted conservation benefits, there are instances where such efforts fall short of achieving desired goals of establishing and/or restoring populations^10^. Guidelines incorporating adaptive conservation measures and information on key biological and ecological aspects of target species and monitoring have proven crucial for identifying conservation shortfalls and planning conservation strategies for effective solutions to increase the overall success of such programs^5^. The guidelines to monitor target species alone may not be adequate in situations, when the target species occur sympatrically and the presence of sympatric species could induce competition impeding the intended recovery of the target species^26,30^. Therefore, information on the status of sympatric species could provide valuable insights to guide program outcomes. In the present study, we assessed the population trend, occupancy and genetic diversity to understand the status of mugger in Chambal River.

Our findings revealed an increase in the population trend and high area of occupancy with moderate level of genetic diversity in the mugger population. This confirms colonization of NCS by mugger, though active management was discouraged. The successful mugger colonization is likely to induce competition and interspecific aggression, which may negatively impact the gharial survival imposing a threat to the long-term conservation effort in the Chambal River. Similar reports of increase in mugger in SGWS confirms that mugger now rapidly colonizing prime gharial habitats. Their increase might have been a key contributing factor to the escalated interspecific aggression between gharials and mugger, ultimately leading to the competitive dominance of the mugger^23,28^. This also rationalise the initial decision of debarring translocation of mugger in gharial-centric riverine protected areas^31^. As a result, this validates the need for extensive monitoring of the mugger along with gharial to develop a robust road map for the recovery of gharial^22^.

### Population trend

Abundance indices provide a valuable tool to assess population trends e.g., increasing, declining, or stable over extended time periods, enabling insights into population dynamics. It is crucial for understanding the status and trajectory of a species, as well as for making informed conservation and management decisions. The population dynamics of recovered populations are often estimated using time-series data over a short period, which lacks the statistical ability to predict the linear population trend accurately and hence may be erroneous^32^. In this study, we used 17 year-long continuous time-series data to obtain reliable estimates of population trends in the mugger population in the NCS. Similar to other crocodilian species, we have also observed an age-structured size class composition within the mugger population^33^, where adult size class constituted the highest percentage (Supplementary Table 1). The population growth analysis showed an increasing trend in the mugger population. Although a ‘one-species one area’ based targeted conservation approach was adopted to restrict interspecific competition, 28 muggers were released in the Sanctuary in 1984^21^. The primary focus of the translocation program was to re-establish a self-sustaining gharial population. However, the mugger population witnessed a significant increase, surging from 19 individuals in 1984 to 674 individuals in 2019 (Supplementary Table 1). The increase in the mugger population can be attributed to the protected nature of the Chambal River and its ecological generalist traits, i.e. broader dietary choice, habitat versatility, and resilience towards environmental fluctuations. In addition to the NCS in the Chambal River, mugger population in other riverine protected areas, such as Katerniaghat Wildlife Sanctuary in the Girwa River, Satkosia Gorge Wildlife Sanctuary in the Mahanadi River and the Corbett National Park in the Ramganga River has shown a considerable increase in numbers and breeding activity in the past^31,34,35^. Population dynamic studies on various crocodylians have revealed differential trends, with some species exhibiting increasing trends while others have shown a decline in population over the study period^36,37^. These trends are often linked to the conservation efforts invested to safeguard the species and the level of threats. Additionally, the timeframe and spatial scale of the study can also impact the observed trends. Therefore, comprehensive and long-term monitoring efforts coupled with effective conservation strategies are crucial for ensuring crocodilian populations' long-term viability and sustainability.

### Single-season site occupancy

The estimated mugger occupancy across the study area indicates that the species is faring well in the Chambal River. In the last couple of decades, they have grown in numbers, as shown by the population trend, and so has their spread, as evidenced by the naïve occupancy (0.88 ± 0.04). The National Chambal Sanctuary within the Chambal River is the longest riverine protected area in India, providing considerable space and suitable protective measures for mugger populations to grow in numbers and expand. The generalist species with high dispersal capacities and broader dietary choices as observed in mugger are less constrained by habitat are expected to colonize sub-optimal habitats^38^.

We observed a strong influence of river width on detection probability (p) wherein the detectability of the mugger decreased with an increase in river width. This may be attributed to the boat surveys being conducted along the mid-river channel, and in the broader channel, the distance between the observer and the river bank increases, hindering the observer's ability to detect mugger individuals basking on the river bank. Similarly, river depth strongly influenced mugger occupancy, which increased till the mean river depth (4.7 m) and saturated after that. The preferred depth of the sympatric species, gharial, in the same riverscape was ≥ 4 m^17^. The river depth preference is possibly related to food availability, movement, and cover from predators. Previous studies have indicated that mugger prefer basking on open river banks with a rocky substrate^39^. Also, their presence in human-dominated riverscapes and water bodies in urban landscapes suggests their remarkable adaptability^40^. Considering its preference and adaptability, we anticipated a positive association with rocky substrate and minimal influence of disturbances on mugger occupancy. However, none of the candidate models (ΔAIC ≤ 2) included rocky substrate as the best predictor variable. Instead, the best-predicted model indicated that sandy substrate was among the associated covariates. This highlights that sandy substrate may be more influential in determining mugger occupancy than previously anticipated. The positive association with sandy substrate type might be because the crocodile prefers an open surface for basking. Mugger may use rocky substrate in the absence of other substrate types, such as sand and clay. This also indicates that species preference for substrate might as well rely on relative availability and suitability when alternative substrate options are limited or absent. The absence of any significant influence of anthropogenic factors on mugger occupancy aligns with the species' adaptability in riverscape with high anthropogenic disturbances. This could be attributed to the generalist nature of the species, where they do not exhibit strict habitat preferences. Unsurprisingly, disturbances resulting from human activities have minimal impact on their occupancy. This further supports the notion that the mugger is highly adaptable and can persist in various environments, including those influenced by high human presence. Unlike this, the saltwater crocodile and American alligator (*Alligator mississippiensis*) were shown to occupy areas with low human interference^36,41^.

### Genetic assessment

The overall genetic diversity for the species in the Chambal River showed moderate values (Table 3). The level of genetic diversity in the mugger population is higher than the gharial population when estimated using a similar set of microsatellite loci^16^. This could be attributed to species life-history traits and widely distributed species retaining high genetic diversity^42–44^. In the absence of studies on the movement of the muggers in Chambal and its tributaries, the possibility of the dispersion of muggers from the tributaries of Chambal contributing to the increasing population and gene-flow to maintain the genetic diversity cannot be ruled out.

The population structure suggests the presence of three genetic units among the four nesting sites (Figs. 2 and 3). The identified genetic units followed a pattern where individuals from nesting sites situated at a short distance (~ 10 km; BR-NG) from each other were assigned to a single cluster and did not show a significant level of genetic differentiation, whereas individuals from sites situated at longer distances (> 100 km) were assigned to separate clusters and showed significant genetic differentiation.

The limited long-distance movement probably explains the observed differentiation pattern observed in released muggers^21^ and might be due to the species life-history traits, such as philopatry, observed in crocodiles^45,46^. As an ecological generalist, the mugger can efficiently utilize resources in a given habitat without dispersing across longer distances in search of optimal basking, nesting, and foraging habitats. Hence, the presence of multiple genetic clusters within Chambal River sites may have been due to the limited movement of the individuals across sites. Since muggers are adapted to move on land with agility, the possibility of obstructing movement due to natural and artificial barriers is very low. The current mugger population in the Chambal River was recovered from an initial population of 33 individuals of all size classes in 1984–1985^18,21^. The *M* ratio and HET in the mugger population confirmed the genetic bottleneck. Since, the crocodile population across India suffered a severe decline in its range during the late-1960s our result corroborates with the past demographic decline in the mugger population.

The utilization of cross-species microsatellite markers is likely to introduce ascertainment bias. Hence, the results should be interpreted with discretion. Further, it is required to increase the number of samples and utilize High Throughput Sequencing techniques, such as Single Nucleotide Polymorphisms (SNPs), to substantiate the findings of the study and gain insights into the fine-scale population genetic structure, timing of occurrence, and magnitude of the bottleneck experienced by the mugger population.

## Conclusions

There has been a pressing need for a scientifically supported evaluation of the conservation efforts for gharials. In the past, no attempts were made to gather comprehensive information on all facets recommended under monitoring guidelines that are essential for the evaluation of the ongoing conservation translocation program. The combination of increasing population trends, widespread distribution, and a moderate level of genetic diversity hint toward the successful colonisation of mugger in the Chambal River and pose serious threat to the recovery of gharial. The rapid colonization of mugger also indicates that mugger is benefitting from gharial specific conservation measures such as habitat protection, nest protection implemented in the National Chambal Sanctuary. In the future, continuous monitoring and follow-up assessments of key facets, including population dynamics and genetic status of the gharial as well as the sympatric mugger, is essential for developing adaptive management strategies to minimize potential conflict from interspecific competition and secure the gharial population in Chambal and other rivers. For safeguarding the gharial population in Chambal River, the strategies should aim at developing mechanisms to control mugger population growth in prime gharial habitats. For instance, by translocating breeding individuals and/or nest to a suitable habitat conducive for mugger. Further, studies to fill knowledge gaps are important to prepare adaptive management plans in the absence of empirical evidence on ecological aspects of the mugger, such as size-class-specific survivability, nest count, and interspecific interactions between gharial and mugger. Further, the increasing mugger population in Chambal and other North Indian rivers needs to be studied to understand the dynamics of interspecies competition and develop differential management strategies where muggeroccur sympatrically with the gharial.

## Methodology

### Study area, permits, and ethical considerations

The Chambal River originates in the Vindhyan Range in Mhow, Indore, Madhya Pradesh, and traverses a course of around 965 km up to its confluence with the Yamuna River in Uttar Pradesh, India^47^. Since 1979,  ~ 600 km stretches of the Chambal River between Kota barrage in Rajasthan and Chambal-Yamuna confluence in Uttar Pradesh, and  ~ 60 km stretches of the Parbati River in Madhya Pradesh (South bank) between Badodiya Bindi and Pali have been protected as National Chambal Sanctuary for the conservation and management of aquatic fauna (Fig. 1).

The Forest Department of Madhya Pradesh (Letter No. 8200 and 2545), Rajasthan (Letter No. 1399), and Uttar Pradesh (Letter No. 3093) provided the necessary permissions for the surveys and collection of the biological samples. No humans were involved during the experiments, and animal ethical clearance was not required as all biological samples were collected from dead hatchlings or hatched eggshells and no animal was captured or handled for sample collection.

### Data collection

#### Population monitoring

The river stretch from Pali (confluence of Chambal-Parvati) to Panchnada (the endpoint of National Chambal Sanctuary) was surveyed annually during the winter months (January–February) of 2017–2019 (Supplementary Fig. 6). We used the total count method to estimate the RDI of mugger i.e. individuals sighted per km of river stretch^48^. The survey was conducted during daylight between 1000 and 1600 h. A motorboat fitted with a 25HP outboard engine was used to survey the river, and the speed of the boat was maintained between 8 and 10 km/hr. Two observers equipped with 8 × 40 mm binoculars were positioned to spot the basking muggers^15^. The mugger was identified based on their morphology using established description keys following the identification guide^49^. When spotted, the number of mugger individuals, visually estimated size of the mugger and associated habitat variables were recorded for each sighting. The observed mugger crocodiles were categorized into size classes following Khadka et al.^50^: Hatchlings (< 30 cm), Yearlings (30 < 50 cm), Juveniles (50 < 125 cm), Sub-adults (125 < 180 cm), and Adults (> 180 cm).

#### Single-season site occupancy

We used spatial replicates in a linear system to determine the drivers of mugger distribution in the Chambal River using single-season site occupancy modeling^51^. We surveyed ~ 400 km in a total of 134 composite blocks, each composite block consisting of three one km spatially replicated blocks. Only survey data for the year 2019 was used for site occupancy analysis. The presence-absence of the mugger was recorded in each block along with associated detection and site covariates (Supplementary Table 6). The covariates were selected based on a priori understanding of crocodylian ecology and literature^39,52^, and assumed to be essential in determining their occupancy and detection probability. A detection history matrix was built using the sightings as presence locations to model the occupancy of the mugger. The detection covariates are thought to potentially influence the probability of detecting a species given the presence in a site (p), and site covariates influence the probability of occupancy (Ψ).

#### Genetic assessment

Biological samples for genetic assessment were collected from  ~ 400 km stretch of the Chambal River during 2017 and 2018 (Fig. 1). The biological samples include tissue obtained from dead remains of hatchlings and chorioallantoic membrane from hatched eggshells. We collected 122 biological samples, including tissue (n = 22) and eggshells containing chorioallantoic membrane (n = 100), from five nesting sites (Pali, Baroli, Nadigaon, Dangbasai, and Tigri) (Supplementary Table 3). The samples were collected opportunistically during post-nesting surveys. The eggshells were kept in a sterile container and air-dried at room temperature, and tissue samples were stored in absolute ethanol at room temperature and later at  -20 °C in the laboratory for long-term storage. A significant portion of the samples collected for genetic assessment were dry eggshells and tissue from dead hatchlings; hence clutch information on these samples is not available. Total genomic DNA was extracted from tissue (80–100 mg) and chorioallantoic membrane (cotton swab) following the phenol–chloroform method^53^. DNeasy blood and tissue kit (Qiagen Inc. USA) were used for samples that failed to recover DNA using phenol–chloroform method.

We used a panel of 10 microsatellite loci previously developed in other crocodile species^54–58^. The loci were selected based on a screening of 36 microsatellite loci developed across 10 species (Supplementary Table 7). Polymerase chain reactions (PCR) were performed following the universal primer-multiplex method^59,60^. The amplified PCR products were subjected to fragment analysis in Applied Biosystems Genetic Analyser, ABI 3500xl as per the manufacturer's protocol with the GeneScan 500 LIZ as a size standard. The alleles were scored using the automated allele scoring feature of GeneMarker v2.7.4 (SoftGenetics, LLC, State College, PA, United States), and finally validated through visual inspection. To produce reliable multilocus genotypes from noninvasive samples, a multiple-tube method was used with three replicates of each sample^61^.

### Data analyses

#### Population trend

The population trend was estimated using contiguous RDI data from 2003 to 2016^31^ and from 2017 to 2019. Altogether we have used time-series data over 17 years to obtain population trend estimates in the mugger population in the Chambal River (Supplementary Fig. 1). The population trends were calculated using the exponential rate of increase (r), the annual finite population growth rate (λ), and the percentage change in population over time^48,62^. We estimated the exponential rate of increase (r) as the slope of the linear regression of RDI transformed to natural logarithms (log_e_) and years^62,63^. The annual finite population growth rate (λ) was estimated by back transformation (λ = er, where e is the base of natural logarithms, ~ 2.71828, and r is the exponential rate of increase). The percentage change was calculated here from mean λ; if λ = 1.15, the population increases at 15% per year.

#### Single-season site occupancy

We used the site occupancy modeling to derive the occupancy of mugger and factors influencing their occupancy in the Chambal River. The analysis was performed using *unmarked* package^64^ implemented in R v4.1.2^65^. Each pair of covariates were tested for spatial correlation using Spearman’s correlation, continuous variables were standardised and categorical variables were provided as a factor for the analysis.

We opted for a two-step approach to model the probability of detection (p) and occupancy (Ψ) as a function of detection and site covariates, respectively^66^. Initially, we built univariate detection (p) model, holding the site covariates constant. The detection models were then ranked based on AIC, and the model corresponding to the lowest AIC was selected. Finally, we used an additive combination of site covariates with selected detection covariates from the previous step to produce the final set of models. Again, these models were ranked according to AIC and models below Δ2 AIC were considered important for predicting the mugger occupancy. We performed the MacKenzie and Bailey goodness of fit test for single-season occupancy models based on the best models to assess the goodness of fit and over dispersion^67,68^. The relative contribution of each variable was assessed using summed Akaike weights^69^. The covariates were considered significant when the estimated beta coefficient at 95% confidence interval did not overlap with zero. We predicted the detection and occupancy probabilities were estimated for each site via model averaging of the final model set using *MuMin* package^70^ in R v4.1.2^65^.

#### Genetic assessment

The genotype of each individual was obtained through the consensus of three replicates, and the quality index was estimated for each locus genotyped following Miquel et al.^71^. The average amplification success was calculated as a percentage of positive PCR amplification against the total number of PCR. The genotyping errors rate per locus was estimated manually following Pompanon et al.^61^, null alleles frequency was estimated using FreeNa^72^ and large allele dropout using Micro-Checker v2.2.3^73^.

PID (unbiased) and PID (sibs) was calculated using Gimlet v1.3.3^74^ and deviations from Hardy–Weinberg equilibrium (HWE) for each locus using GenAlEx v6.51^75^. The genetic diversity estimates (the number of alleles per locus, observed heterozygosity, and expected heterozygosity) were estimated using Arlequin v3.0^76^, and allelic richness was estimated using a rarefaction approach implemented in HP-Rare v1.1 to account for the uneven sample size across nesting sites^77^.

The genetic differentiation indices *F*_ST_^78^ and Jost’s *D*^79^ were estimated in R studio 2023.03.1^65^ using the *strataG* package^80^ with 10^3^ bootstrap iterations. The population genetic structure was inferred using two approaches: (a) Systematic model-based Bayesian clustering approach that uses allele frequencies at each locus to infer the population structure implemented in Structure v2.3.4^81^. The analysis was performed for 1 to 10 clusters (K). For each K, 10 iterations were run under the admixture with correlated allele model. The simulations were run for 10^5^ burn-in and 10^6^ Markov chain Monte Carlo iterations (MCMC). The optimum number of K was inferred using delta K^82^, which was estimated using the web version of Structure Harvester, v0.6.94^83^. The assignment plot was prepared using the program Distruct v1.1^84^. (b) Discriminate Analysis of Principal Component (DAPC), a multivariate non-model-based approach to identify and describe genetic clusters^85,86^. The analysis was performed using the adgenet in R studio 2023.03.1^65^. The optimal number of clusters in DAPC was estimated based on the lowest associated BIC.

We examined the evidence of a genetic bottleneck using two different approaches. First, the heterozygosity excess test approach was performed in Bottleneck v1.2.02^87^. A one-tailed Wilcoxon test was used to determine the presence of a significant number of loci with excess heterozygosity to confirm the presence of a bottleneck. The estimates were calculated under two mutation models: Single-step Mutation Model (SMM) and Two-phase Mutation Model (TPM). TPM tends to be the most appropriate mutation model for microsatellite loci^88^. TPM was carried out at 95% SMM (variance at 12), and the simulations were run for 104 iterations^87^. Second, we used the Garza-Williamson index (or *M* ratio) implemented in Arlequin v3.1^76^. The *M* ratio estimates the ratio of the observed number of alleles to the size of the allele range based on the assumption that the ratio is expected to decrease due to the random loss of alleles in a recently reduced population. The calculated *M* ratio was then compared with the critical value (*M*_C_ = 0.68). An *M* ratio below *M*_C_ is considered a genetic bottleneck signature^89^. Due to fewer samples than recommended to obtain a reliable result for evidence of genetic bottleneck in two of three identified genetic clusters, the tests were performed by pooling samples together.

### Supplementary Information

Supplementary Information.

## Data Availability

The datasets generated and/or analysed during the current study are available from the corresponding author upon reasonable request.
