# Supplementary material for: Population status and genetic assessment of mugger (Crocodylus palustris) in a tropical regulated river system in North India
Source: Sci Rep. 2024 Mar 28;14:7438. doi: 10.1038/s41598-024-57983-2 (PMC10978964; doi:10.1038/s41598-024-57983-2)
Supplement: Supplementary file 1 — Supplementary Information. [file 41598_2024_57983_MOESM1_ESM.docx]

**Supplementary Information-** Population status and genetic assessment of mugger (*Crocodylus palustris*) in a tropical regulated river system in North India.

Surya Prasad Sharma^1^, Mirza Ghazanfarullah Ghazi^1^, Suyash Katdare^1^, Ruchi Badola^1^ and Syed Ainul Hussain^1^*

^1^Wildlife Institute of India, P.O. Box # 18, Chandrabani, Dehra Dun, 248002, Uttarakhand, India.

***Corresponding author** - Syed Ainul Hussain, E-mail: [ainul.hussain@gmail.com](mailto:ainul.hussain@gmail.com) http://orcid.org/0000-0003-3229-806X

Surya Prasad Sharma – suryapdsharma@gmail.com, https://orcid.org/0000-0002-7411-4284,

Mirza Ghazanfarullah Ghazi – mirzaghazanfarullah@gmail.com, https://orcid.org/0000-0003-4737-2170

Suyash Katdare – suyashk18@gmail.com, https://orcid.org/0000-0003-4884-6761

Ruchi Badola – [ruchi@wii.gov.in](mailto:ruchi@wii.gov.in), https://orcid.org/0000-0001-7124-5134

Syed Ainul Hussain – [ainul.hussain@gmail.com](mailto:ainul.hussain@gmail.com), http://orcid.org/0000-0003-3229-806X

**Supplementary Figures**

**Supplementary Fig. 1**: Mugger numbers and Relative Density Index (RDI) in last 17 years in National Chambal Sanctuary within Chambal River between 2003 and 2019.

**Supplementary Fig. 2**: Relationship between ln RDI of mugger and year in the National Chambal Sanctuary within Chambal River between 2003 and 2019.


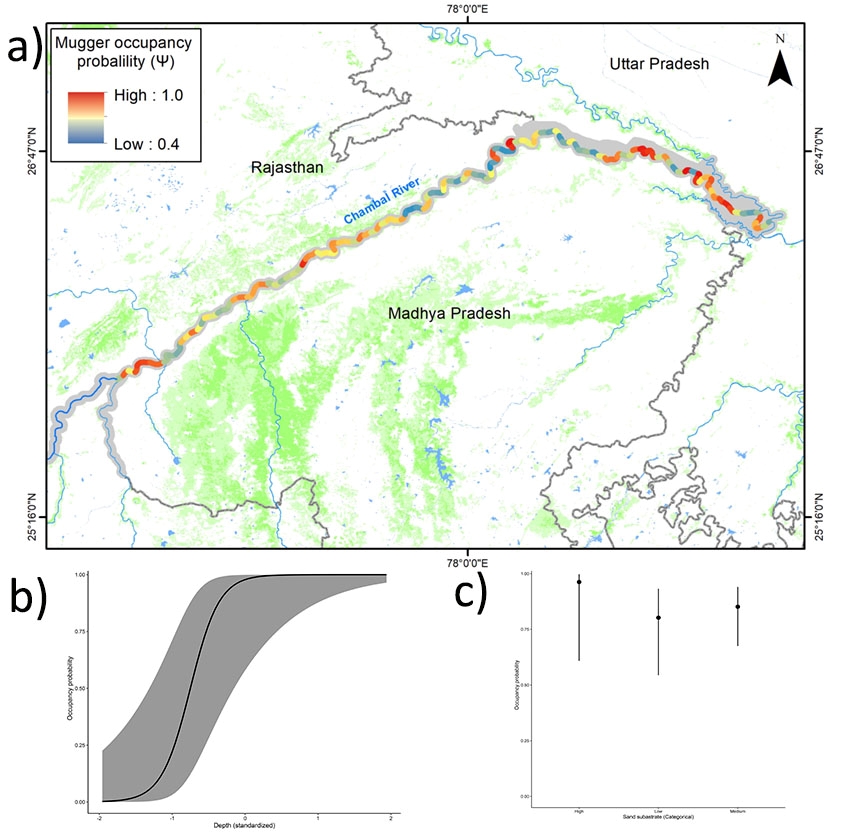
**Supplementary Fig. 3**: Map showing modelled averaged prediction for mugger (a) occupancy probability (Ψ) in the National Chambal Sanctuary within Chambal River and plots of best predictor covariates for mugger occupancy against (b) depth and (c) substrate type sand. The map was prepared using ArcGIS v.10.3.1 software developed by ESRI (https://www.esri. com) and plots were prepared in RStudio 2023.03.1 (https://posit.co/download/rstudio-desktop/) using package *ggplot2* (https://www.rdocumentation.org/packages/ggplot2/versions/3.4.2).


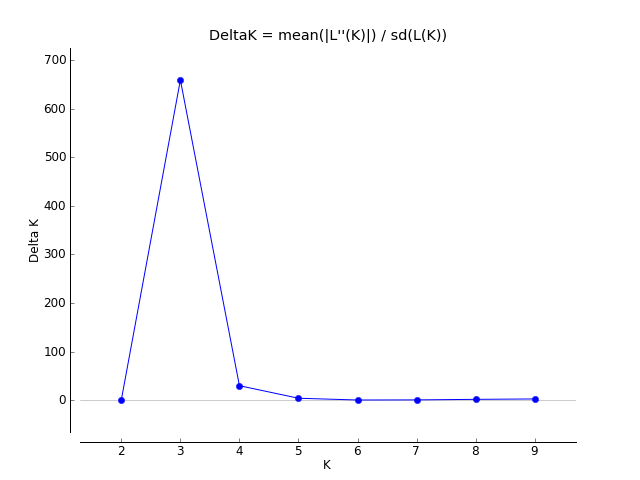
**Supplementary Fig. 4**: The plot of delta *K* obtained using web version of program Structure harvester.


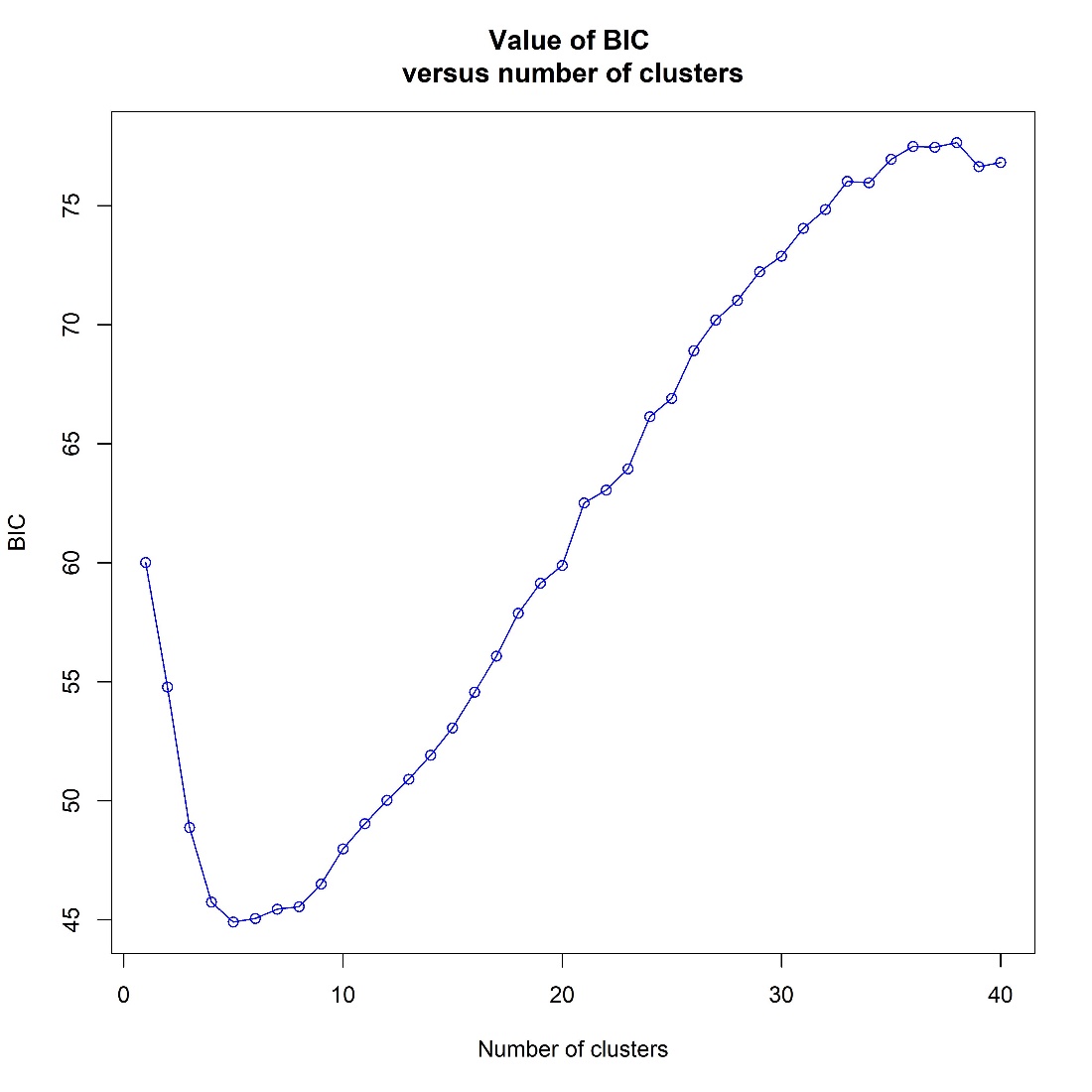
**Supplementary Fig. 5**: The BIC plot obtained from DAPC analysis.


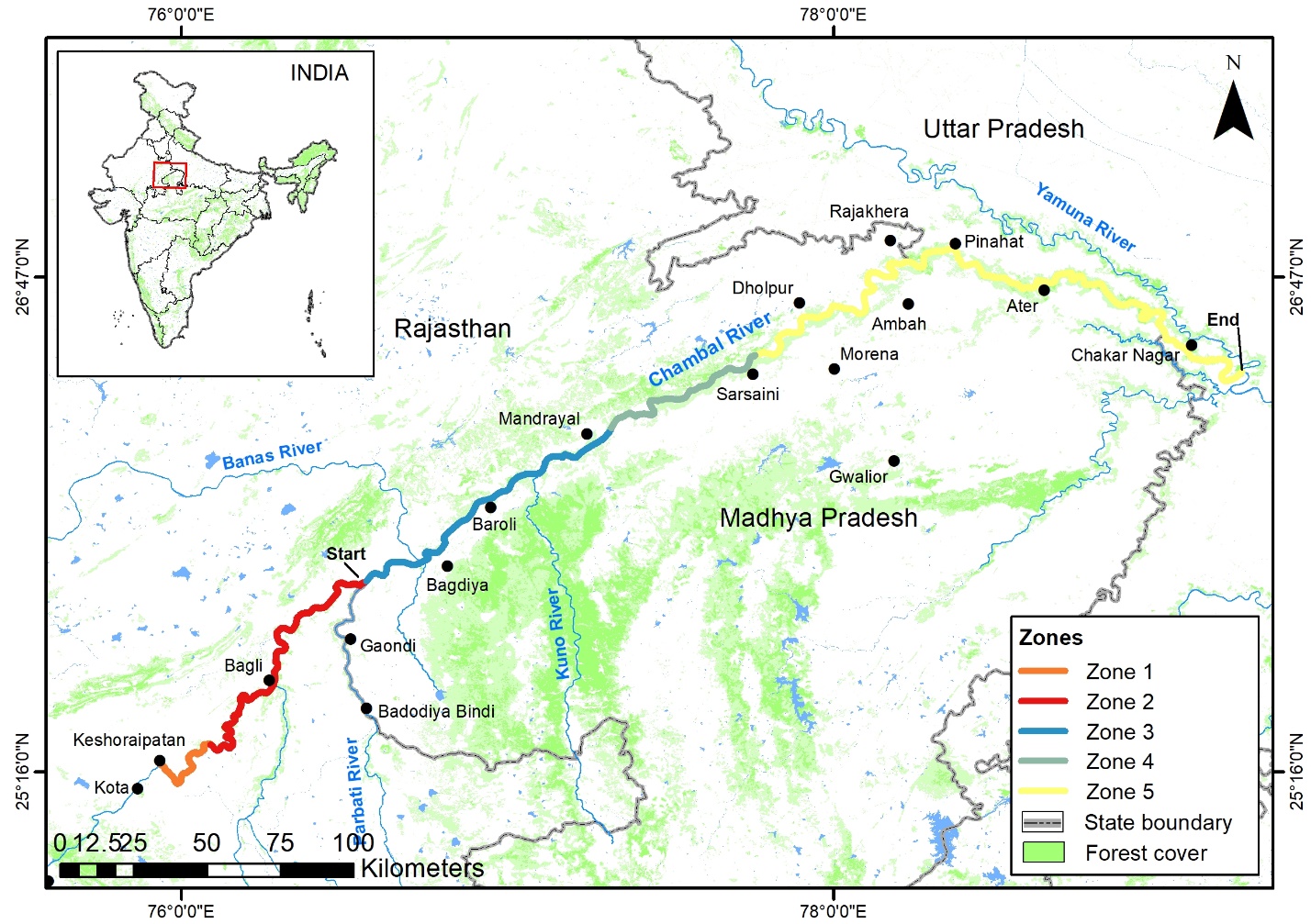
**Supplementary Fig. 6**: Map showing five zones in the Chambal River categorized by Singh et al 1985. The population census survey was conducted in lower three zones (Zones 3 -Zone 5) ~400 km. The map was prepared using ArcGIS v.10.3.1 software developed by ESRI (https://www.esri. com).

**Supplementary Tables**

**Supplementary Table 1:** Summary of the total number and size class of mugger observed during population census surveys during 2017-2019 in the National Chambal Sanctuary within Chambal River. Adult male (AM), other adults (A), subadult (SA), juvenile(J), yearling (Y), and hatchling (H). The percent composition of each size class is given in parentheses.

| **Year\Size Class** | **A**  **(>180 cm)** | **SA**  **(125<180 cm)** | **J**  **(50<125 cm)** | **Y**  **(30<50 cm)** | **H**  **(<30 cm)** | **Total** |
| --- | --- | --- | --- | --- | --- | --- |
| **2017** | 465 (82.74%) | 71 (12.63%) | 23 (4.09%) | 2 (0.36%) | 1 (0.18%) | **562** |
| **2018** | 512 (81.08%) | 55 (8.65%) | 30 (4.40%) | 7 (1.14%) | 8 (1.31%) | **592** |
| **2019** | 604 (84.36%) | 53 (7.40%) | 17 (2.37%) | -- | -- | **674** |

**Supplementary Table 2**: Parameter estimate variables of the top model for mugger site occupancy. ‘*’ indicates variables with significant influence on mugger site occupancy.

| **Model** | **p(temp)**  **β±SE (95% CI)** | **p(width)**  **β±SE (95% CI)** | **Ψ(depth)**  **β±SE (95% CI)** | **Ψ(mining)**  **β±SE (95% CI)** | **Ψ(sandH)**  **β±SE (95% CI)** | **Ψ(sandL)**  **β±SE (95% CI)** | **Ψ(sandM)**  **β±SE (95% CI)** | **Ψ(width)**  **β±SE (95% CI)** |
| --- | --- | --- | --- | --- | --- | --- | --- | --- |
| p(width), Ψ(depth + sand) | -- | -0.36 **±** 0.12 *  (-0.6 - -0.13) | 5.06 **±** 1.91 *  (1.32 - 8.79) | -- | 2.12 **±** 1.24  (-0.31 - 4.55) | -0.46 **±** 1.42  (-3.24 - 2.32) | 0.5 **±** 1.14  (-1.74 - 2.74) | -- |
| p(width), Ψ(depth + mining) | -- | -0.38 **±** 0.12 *  (-0.61 - -0.15) | 3.85 **±** 2.2  (-0.46 - 8.17) | -0.55 **±** 0.38  (-1.3 - 0.19) | -- | -- | -- | -- |
| p(width), Ψ(depth) | -- | -0.37 **±** 0.12) *  (-0.6 - -0.14) | 4.52 **±** 1.72 *  (1.16 -7.89) | -- | -- | -- | -- | -- |
| p(width), Ψ(depth + width) | -- | -0.4 **±** 0.12 *  (-0.64 - -0.17) | 4.12 **±** 1.73 *  (0.73 - 7.51) | -- | -- | -- | -- | 0.68 **±** 1.01  (-1.29 - 2.65) |
| p(temp + width), Ψ(depth) | 0.11 **±** 0.11  (-0.12 - 0.33) | -0.34 **±** 0.12 *  (-0.58 - -0.1) | 4.44 **±** 1.74 *  (1.02 - 7.85) | -- | -- | -- | -- | -- |

**Supplementary Table 3**: Information on site wise samples collected for genetic analysis, sample type, number of samples genotyped, and number of unique samples obtained using multilocus genotype data. *Sample from Dangbasai nesting site was excluded from the analysis.

| **Location** | **Latitude** | **Longitude** | **No. of samples collected** | **No of sample genotyped** | **No. of unique samples** |
| --- | --- | --- | --- | --- | --- |
| Pali | 25.8444 | 76.5617 | 14 | 10 | 6 |
| Baroli | 26.1036 | 76.9432 | 18 | 17 | 14 |
| Nadigaon | 26.1428 | 77.0031 | 69 | 39 | 26 |
| Dangbasai* | 26.5407 | 77.7578 | 6 | 1 | 1 |
| Tigri | 26.6927 | 78.0035 | 15 | 14 | 13 |
| **Total** |  |  | **122** | **81** | **60** |

**Supplementary Table 4**: Summary of amplification success, quality index, error rate per locus and null allele frequency across ten loci examined.

| **Locus name** | **Amplification**  **success** | **Quality Index** | **Error rate**  **per locus** | **Null allele**  **frequency** |
| --- | --- | --- | --- | --- |
| CpPSSR14 | 86.83 | 0.95 | 0.05 | 0.049 |
| CpPSSR12 | 97.12 | 0.92 | 0.08 | 0.000 |
| CUD68 | 84.36 | 0.88 | 0.12 | 0.000 |
| 4HDZ27 | 94.65 | 0.92 | 0.08 | 0.000 |
| 4HDZ391 | 92.59 | 0.90 | 0.10 | 0.000 |
| G13_2 | 85.60 | 0.94 | 0.06 | 0.000 |
| G13_14 | 85.19 | 0.95 | 0.05 | 0.024 |
| G13_18 | 85.60 | 0.96 | 0.04 | 0.009 |
| CpP203 | 85.60 | 0.95 | 0.05 | 0.000 |
| CpP208 | 87.65 | 0.96 | 0.04 | 0.058 |
| **Mean** | 88.61 | 0.93 | 0.07 | 0.01 |
| **SE** | 1.44 | 0.008 | 0.008 | 0.007 |

**Supplementary Table 5**: The genetic diversity estimates of 59 individuals of muggers in the National Chambal Sanctuary within Chambal River (PA-Pali, BR-Baroli, NG-Nadigaon and TG-Tigri). Number of polymorphic loci (Pl); number of samples (N); number of alleles per locus (Na); standardised allelic richness (Ar); observed heterozygosity (Ho) and expected heterozygosity (He). The values in the parentheses are standard deviation for each locus.

| Nesting Sites | Pl | N | Na | Ar | Ho | He |
| --- | --- | --- | --- | --- | --- | --- |
| PA | 8 | 6 | 2.1 | 2.1 | 0.40 | 0.39 |
| BR | 10 | 14 | 3.9 | 3.2 | 0.59 | 0.55 |
| NG | 9 | 26 | 3.8 | 3.1 | 0.66 | 0.55 |
| TG | 8 | 13 | 2.9 | 2.6 | 0.47 | 0.43 |
| Overall |  | 59 | 3.18(0.85) | 2.75(0.51) | 0.53(0.12) | 0.48(0.08) |

**Supplementary Table 6**: List of detection and site covariates against which mugger occupancy and detection probability were modeled in single-season occupancy framework.

| **Covariates** | **Type** |
| --- | --- |
| **Detection** |  |
| Time | Continuous (hour: minutes) |
| Temperature | Continuous (◦ C) |
| River width | Continuous (meter) |
| **Site** |  |
| River depth | Continuous (meter) |
| River width | Continuous (meter) |
| Substrate type | Categorical (S: sandy, C: clay, R: rocky) |
| Fishing | Categorical (Number of fishing gears in each block) |
| Sand mining | Categorical (Number of mining in each block) |

**Supplementary Table 7**: List of microsatellite loci screened for the genetic assessment. ^1^Polymorphic; ^2^No amplification; ^3^Monomorphic; and ^4^Inconsistent allele.

| **S. No.** | **Locus name** | **Species** | **Repeat motif** | **Reference** |
| --- | --- | --- | --- | --- |
| 1 | CpSSR14 | *C. palustris^1^* | DI | Aggarwal et al. 2014 |
| 2 | CpSSR12 | *C. palustris^1^* | DI | Aggarwal et al. 2014 |
| 3 | CUD68 | *C. acutus^1^* | DI | FitzSimmons et al. 2000 |
| 4 | Cj16 | *C. johnstoni^1^* | DI | FitzSimmons et al. 2000 |
| 5 | 4HDZ27 | *C. mindorensis^1^* | DI | Hinlo et al. 2014 |
| 6 | 4HDZ391 | *C. mindorensis^1^* | DI | Hinlo et al. 2014 |
| 7 | G13_2 | *G. gangeticus^1^* | TETRA | Jogayya et al. 2013 |
| 8 | G13_14 | *G. gangeticus^1^* | TETRA-DI-TETRA | Jogayya et al. 2013 |
| 9 | CpP203 | *C. porosus^1^* | TETRA | Miles et al. 2008 |
| 10 | CpP208 | *C. porosus^1^* | TETRA | Miles et al. 2008 |
| 11 | G13_7 | *G. gangeticus^2^* | TETRA | Jogayya et al. 2013 |
| 12 | CpP107 | *C. porosus^2^* | TETRA | Miles et al. 2008 |
| 13 | CpP121 | *C. porosus^2^* | TETRA | Miles et al. 2008 |
| 14 | G13_5 | *G. gangeticus^2^* | TETRA | Jogayya et al. 2013 |
| 15 | G13_6 | *G. gangeticus^2^* | TETRA | Jogayya et al. 2013 |
| 16 | G13_8 | *G. gangeticus^2^* | TETRA | Jogayya et al. 2013 |
| 17 | Cc_D07 | *C. crocodilus^2^* | DI | Oliveira et al. 2010 |
| 18 | Cc_E08 | *C. crocodilus^2^* | DI | Oliveira et al. 2010 |
| 19 | CpDi04 | *C.porosus^2^* | DI | Miles et al. 2008 |
| 20 | CpP3220 | *C. porosus^2^* | TETRA | Miles et al. 2008 |
| 21 | CpP722 | *C. porosus^2^* | TETRA-DI | Miles et al. 2008 |
| 22 | G13_9 | *G. gangeticus^3^* | DI-TETRA-DI | Jogayya et al. 2013 |
| 23 | CpP1401 | *C. porosus^3^* | TETRA | Miles et al. 2008 |
| 24 | CpP309 | *C. porosus^3^* | TETRA | Miles et al. 2008 |
| 25 | TGE2 | *A. sinensis^3^* | DI | Yu et al. 2011 |
| 26 | G13_1 | *G. gangeticus^3^* | TRI-DI-DI | Jogayya et al. 2013 |
| 27 | G13_11 | *G. gangeticus^3^* | TETRA-DI | Jogayya et al. 2013 |
| 28 | G13_15 | *G. gangeticus^3^* | TETRA | Jogayya et al. 2013 |
| 29 | G13_18 | *G. gangeticus^3^* | DI-DI | Jogayya et al. 2013 |
| 30 | CpSSR13 | *C. palustris^4^* | DI | Aggarwal et al. 2014 |
| 31 | CpSSR19 | *C. palustris^4^* | DI | Aggarwal et al. 2014 |
| 32 | 4HDZ35 | *C. mindorensis^4^* | DI | Hinlo et al. 2014 |
| 33 | G13_16 | *G. gangeticus^4^* | TETRA | Jogayya et al. 2013 |
| 34 | Cj119 | *C. johnstoni^4^* | DI | FitzSimmons et al. 2000 |
| 35 | Cy-l14 | *C. yacare^4^* | DI | Godshalk et al. 2006 |
| 36 | TM23 | *T. schlegelii^4^* | TETRA | Chang et al. 2004 |
